# Unsuspected Clenbuterol Toxicity in a Patient Using Intramuscular Testosterone

**DOI:** 10.5811/cpcem.2017.2.33318

**Published:** 2017-05-23

**Authors:** Matthew K. Griswold, Eike Blohm, Roderick Cross, Edward W. Boyer, Jennifer L. Carey

**Affiliations:** * University of Massachusetts School of Medicine, Department of Emergency Medicine, Division of Toxicology, Worcester, Massachusetts; † University of Massachusetts School of Medicine, Department of Emergency Medicine, Worcester, Massachusetts

## Abstract

Clenbuterol is a beta-agonist that has been abused by fitness-oriented individuals for muscle growth and weight loss. We report a case of a 46-year-old man who presented tachycardic, hypokalemic, and hyperglycemic after injecting testosterone obtained from Brazil. He developed refractory hypotension and was started on an esmolol infusion for suspected beta-agonist toxicity. Laboratory analysis showed a detectable clenbuterol serum concentration. Analysis of an unopened ampule contained boldenone undecylenate, clenbuterol, and vitamin E. This case illustrates a novel exposure that caused beta-agonist toxicity and was treated successfully with rapid-onset beta blocker.

## INTRODUCTION

Clenbuterol is a beta-agonist that has been abused by bodybuilders and other fitness-oriented individuals to promote muscle growth and weight loss by stimulating the β_3_ receptor. Clenbuterol in the past has been used as an adulterant for heroin.^1^ Abuse of clenbuterol may produce hemodynamic compromise, life-threatening dysrhythmias and electrolyte disturbance.^2^ Toxicity is easily confused with septic shock, but treatment is diametrically opposed; vasopressor therapy with additional β-agonism is detrimental to patients with clenbuterol toxicity as it will promote further tachycardia and subsequent hemodynamic collapse. Instead, β-antagonists are appropriate therapy for clenbuterol-induced hypotension.

## CASE REPORT

**Image 1:**
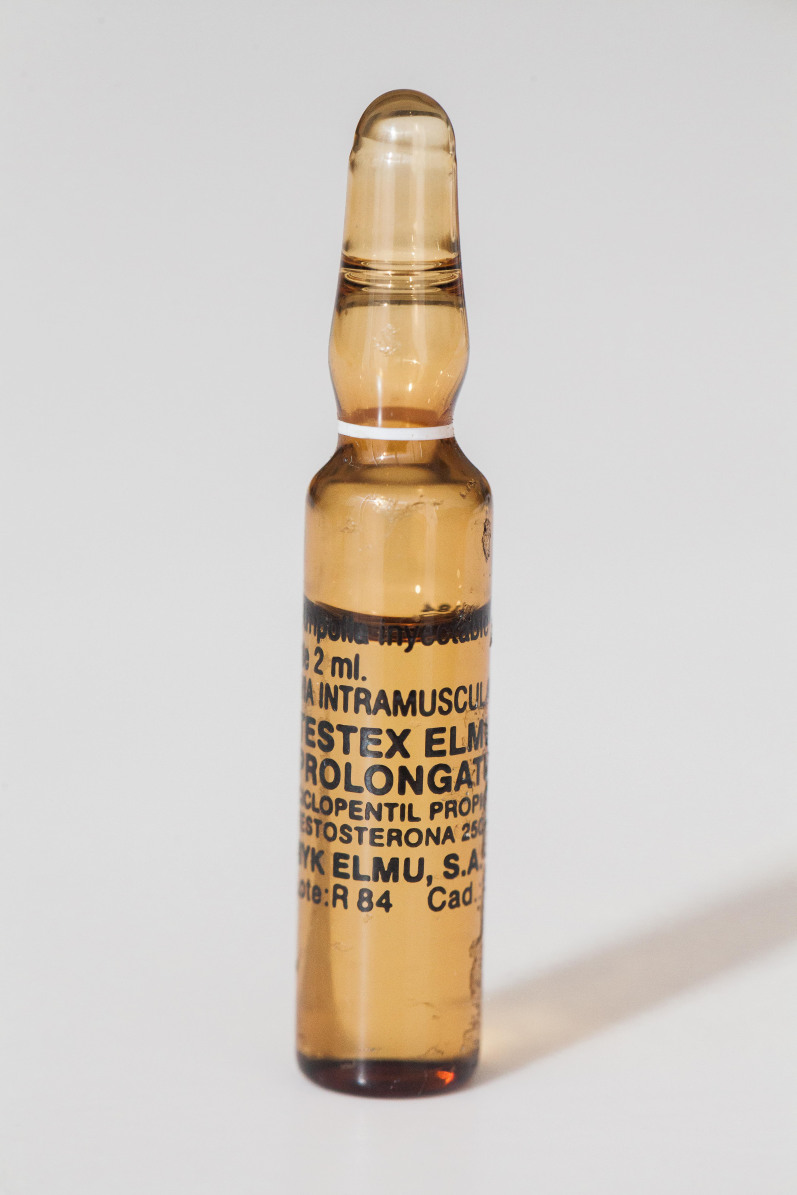
Ampule of substance injected by patient with suspected clenbuterol toxicity.

A 46-year-old man with a past medical history significant only for a traumatic back injury eight years prior, presented to the emergency department (ED) with acute onset of dizziness, nausea, and palpitations. His chronic back pain was refractory to standard anti-inflammatory medications and local corticosteroid injections. Six months prior to our encounter with the patient, he experienced symptom relief from a 1 mL intramuscular (IM) injection of a reported testosterone of unknown concentration, which he had received from a friend. He had near-complete resolution and remained comfortable for a period of several months. The patient reported recurrence of his back pain; thus he decided to again attempt testosterone for pain relief. He self-administered a 2 mL injection of Testex Elmu Prolongatum 250 mg (testosterone cypionate) (Image 1).

The substance was contained in sealed-glass vials that had been purchased from Brazil and imported by a family member into the United States. Within minutes of the injection, he developed symptoms of palpitations, nausea and vomiting. He initially presented to a community hospital; however, he left prior to being evaluated by a physician. His symptoms persisted and the following day he returned to the same ED, where he was observed overnight. Serial troponin levels were negative and he was discharged the following day upon improvement of his symptoms.

**Image 2:**
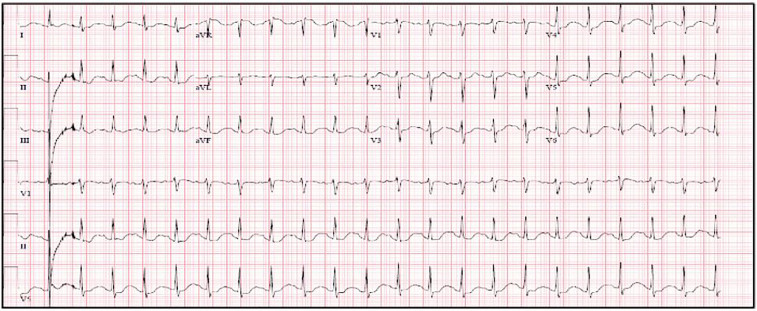
ECG at time of arrival.

Two months following the previous episode, the patient again self-injected intramuscular testosterone. Although product lot numbers were unavailable, the patient reported the vial originated from the same Brazilian shipment. Again, he experienced rapid onset of nausea, vomiting and palpitations. He initially presented to an affiliate hospital where he was found to have a heart rate of 130 beats per minute (bpm) and a blood pressure of 129/66 mmHg. An electrocardiogram (ECG) featured a junctional rhythm with QRS and QTc intervals of 92, and 608 msec respectively, and ST depression in the inferolateral leads indicative of myocardial strain (Image 2).

Labs were remarkable for potassium of 2.6 mmol/L (normal range: 3.5–5.3 mmol/L) and serum glucose of 261 mg/dL. His prolonged QTc was likely due to his hypokalemia. The patient denied history of diabetes mellitus. Serum troponin concentrations were undetectable (<0.02 ng/mL). Out of concern for a possible allergic reaction he was treated with one liter of intravenous (IV) normal saline, 50 mg IV diphenhydramine and 125 mg IV methylprednisolone. He was also administered 80 mEq oral potassium and 2 mg IV lorazepam. Subsequently, the patient developed acute hypotension with blood pressure falling to 70/33 mmHg, and he was given an additional 2.5 liters of normal saline. Fluid resuscitation failed to resolve his hypotension, and the patient was transferred to our tertiary care center for further evaluation and care.

Upon arrival to our facility, the patient’s heart rate was 120 bpm and he remained hypotensive at 105/43 mmHg. We suspected the testosterone preparation contained clenbuterol, and out of concern for β-agonist toxicity he was started on esmolol (0.5 mg/kg bolus followed by a 50 mcg/kg/min infusion). Shortly after titration of esmolol, the patient’s hypotension resolved and his tachycardia improved. He remained on the esmolol drip for a total of 12 hours. At the time of discharge, his heart rate was 98 bpm with a blood pressure of 120/66 mmHg. We recommended that the patient discontinue use of the testosterone product.

Laboratory workup during the patient’s hospitalization included serial troponin serum levels, electrolyte monitoring and toxicology screens. Serial ECGs were performed, and the patient’s rhythm improved to a sinus tachycardia at a rate of 109 bpm and a PR interval of 164 ms. The ST depression resolved. Serial serum troponin concentrations remained normal. A urine comprehensive drug screen (performed by gas chromatography mass spectroscopy at our institution) was positive for caffeine and diphenhydramine. A quantitative caffeine level drawn two hours after the comprehensive drug screen was negative (<1 mcg/mL); hence methylxanthine toxicity was deemed unlikely. Qualitative analysis performed by NMS Laboratories (Willow Grove, PA) showed a positive clenbuterol serum concentration (>10 ng/mL). An unopened glass ampule provided by the patient was sent to NMS’s crime laboratory. It was found to contain boldenone undecylenate, clenbuterol, and vitamin E.

CPC-EM Capsule

What do we already know about this clinical entity?

Beta-agonist toxicity is described in toxicology literature, but it is not well known to other specialties. It may go unrecognized in the ED setting, as it mimics other pathologies such as sepsis.

What makes this presentation of disease reportable?

There are no published reports of clenbuterol adulterated testosterone. It is an important entity to recognize, as clenbuterol adulteration may appear elsewhere, such as to “cut” heroin.

What is the major learning point?

Consider beta-agonist toxicity in patients with tachycardia, hypokalemia and hyperglycemia. Short-acting beta antagonists improve hemodynamic output, particularly in hypotensive patients.

How might this improve emergency medicine practice?

Recognize beta-agonist toxicity (which mimics other common illnesses) and correctly treat it, despite therapy being diametrically opposed to “typical” therapy for tachycardia in a hypotensive patient.

## DISCUSSION

### Differential Diagnosis

The patient in this case presented with tachycardia, hypotension, hyperglycemia, and hypokalemia. In developing the differential diagnosis and treatment plan for this patient, dividing the signs and symptoms into hemodynamic and metabolic categories was an effective approach.^3^ Hemodynamically the patient was tachycardic and hypotensive, while metabolically the patient exhibited hyperglycemia and hypokalemia. This method will lead to distinct differential diagnoses for each category. Subsequently, the potential diagnoses can be compared across categories for similarities.

Toxicological etiologies of tachycardia include sympathomimetics, anticholinergics, hallucinogenics, β-adrenergic agonists, methylxanthines (such as caffeine or theophylline), and drug withdrawal.^4^ Causes of hypotension include alpha-1 adrenergic antagonists (including tricyclic antidepressants), alpha-2 adrenergic agonists such as clonidine, β-adrenergic agonists, nitrates, carbon monoxide, cyanide, opiates, sedative hypnotics, and calcium channel blockers.^5^,^6^ Hyperglycemia can be induced by β-adrenergic agonists, methylxanthines, and calcium channel blockers.^7^,^8^ Moreover, adrenergic agonists, methylxanthines, and diuretics will cause hypokalemia.^9^ Regarding the patient in this vignette, β-adrenergic agonists or methylxanthines explained the hemodynamic and metabolic abnormalities and suggested the likely toxicological etiology. In this presentation, methylxanthine toxicity was less likely as the patient did not have the other manifestations of severe methylxanthine overdose such as protracted emesis, seizure, or altered mental status.

### Pathophysiology

Clenbuterol is an agonist at β_1_, β_2_, and β_3_ receptors. It is not approved for human use in the U.S., but is available in several European countries and Mexico as a bronchodilator (β_2_ agonist) for acute asthma exacerbation.^10^ Daily regimens range from 20–200 mcg given 1–3 times daily. The drug is available as an IV/IM injection as well as an oral formulation with a bioavailability of 70–80%, and an elimination half-life of 25–39 hours.^11^ Clenbuterol is abused by bodybuilders for lean muscle development and to reduce adipose tissue. Abuse of clenbuterol produces rhabdomyolysis, myocardial infarction, as well as life-threatening dysrhythmias and electrolyte disturbance.^2^,^12^ High levels of clenbuterol can cause prolonged tachycardia, hypokalemia, and hypophosphatemia.^2^

The effects desired by bodybuilders are mediated by adipocyte β_3_ receptors, which stimulate lipolysis to reduce adipose tissue, as well as striated muscle β_2_ agonism, which increases muscle mass by nutrient partitioning.^13^ Toxicity from clenbuterol is predominately caused by β_2_ agonism which mediates most of the drug’s toxicity, including tachycardia, peripheral vasodilation, hypokalemia, and hyperglycemia.

In addition to the desired actions of clenbuterol, activation of the adrenergic system also leads to undesired side effects via a β_2_ adrenergic G-protein linked receptor. This results in increased chronotropy, intracellular shift of potassium and subsequently low serum potassium concentrations, and hyperglycemia.^3^,^5^,^10^ Patients with clenbuterol toxicity present with tremor, palpitations, anxiety, shortness of breath, and vomiting.^10^,^14^ Expected vital signs include tachycardia and hypotension. Laboratory workup may show hyperglycemia, hypokalemia, hypophosphatemia, and hypomagnesemia.^2^,^10^ In addition, patients may have leukocytosis and an anion-gap acidosis.^15^

### Treatment

Clenbuterol toxicity can be easily confused with septic shock, but treatment is diametrically opposed. While intravenous crystalloid resuscitation is appropriate in both conditions, vasopressor therapy with additional β-agonism is detrimental to patients with clenbuterol toxicity as it will promote further tachycardia and subsequent hemodynamic collapse. Instead, the hemodynamic changes in clenbuterol toxicity should be treated with β-antagonists.^10^ Short-acting β-antagonists that allow for easy titration, such as esmolol, are preferred. Patients may require treatment of up to 72 hours.^10^ Electrolyte abnormalities should be corrected early in the course of treatment.

Patients who progress to cardiac arrest with ventricular tachycardia or fibrillation should receive chest compressions and defibrillation, but epinephrine boluses should be withheld as they can cause further β-agonism and worsen the patient’s condition. There are no data to support this recommendation apart from the understanding of the pathophysiology in this toxicity.

## CONCLUSION

β-agonist toxicity is an uncommon but potentially dangerous condition that can be associated with the increased use of pharmaceuticals or bodybuilding, increasing fitness, and weight loss.^12^ It has a relatively specific toxidrome that can commonly be overlooked when treating the undifferentiated hypotensive, tachycardic patient. Definitive treatment of β-agonist toxicity is with a β-antagonist; however, this can be counter-intuitive in the treatment of the hypotensive patient. We illustrate a case of a patient presenting with symptoms mimicking other common conditions such as sepsis or anaphylaxis. Emergency clinicians must be astute and consider a variety of alternative diagnoses, which includes β-agonist toxicity, as the treatment for this is diametrically opposed to standard treatments.
